# Recent Advance in Antigen-Specific Immunotherapy for Acute Myeloid Leukemia

**DOI:** 10.1155/2011/104926

**Published:** 2011-10-19

**Authors:** Norimitsu Kadowaki, Toshio Kitawaki

**Affiliations:** Department of Hematology and Oncology, Graduate School of Medicine, Kyoto University, 54 Shogoin Kawara-cho, Sakyo-ku, Kyoto 606-8507, Japan

## Abstract

Relapse after chemotherapy is inevitable in the majority of patients with acute myeloid leukemia (AML). Thus, it is necessary to develop novel therapies that have different antileukemic mechanisms. Recent advances in immunology and identification of promising leukemia-associated antigens open the possibilities for eradicating minimal residual diseases by antigen-specific immunotherapy after chemotherapy. Several methods have been pursued as immunotherapies for AML: peptide vaccines, granulocyte-macrophage colony-stimulating factor-secreting tumor vaccines, dendritic cell vaccines, and adoptive T cell therapy. Whereas immunogenicity and clinical outcomes are improving in these trials, severe adverse events were observed in highly avid engineered T cell therapies, indicating the importance of the balance between effectiveness and side effects in advanced immunotherapy. Such progress in inducing antitumor immune responses, together with strategies to attenuate immunosuppressive factors, will establish immunotherapy as an important armament to combat AML.

## 1. Introduction

The immune system has an exquisite ability to specifically kill cells that express particular antigens. This specificity is the heart of immunotherapies that eliminate tumor cells without damaging normal cells. Recent advances in immunology research have revealed many facets in the immune system that are important to develop tumor immunotherapy. At the same time, recent studies have identified many promising acute-myeloid-leukemia- (AML-) associated antigens that can be targeted by immunotherapy. The combination of such advancement may enable antigen-specific immunotherapies to be established as a viable choice of therapy for AML.

Here we review recent advance in antigen-specific autologous immunotherapy for AML and raise several issues to overcome in order to improve clinical efficacy in the future. This review excludes graft-versus-leukemia (GVL) effects exploited in allogeneic hematopoietic stem cell transplantation, which are mainly allogeneic immune reactions.

## 2. Importance of Immunotherapy for AML

Cytotoxic chemotherapy and allogeneic transplantation are practically the only two modalities to treat AML. Chemotherapy has inevitable limitations of effectiveness due to chemoresistance in the majority of AML patients, except for a small fraction of patients with favorable karyotypes. Allogeneic transplantation is inherently accompanied by a variety of life-threatening complications related to graft-versus-host disease (GVHD), which limits candidates to younger and fit patients. Given such situations, immunotherapy may potentially play an important part in the treatment for AML, mainly from the following two standpoints. 

### 2.1. Treatment of Minimal Residual Disease after Chemotherapy

Although it is possible to achieve complete remission by a series of initial chemotherapies in about 80% of AML patients, recurrence is inevitable in the majority of the patients without allogeneic transplantation. It has been reported that leukemia stem cells are resistant to chemotherapy and that it may be an important reason why it is difficult to eradicate AML cells in the majority of patients [1]. This necessitates the development of novel therapies that have different antileukemic mechanisms. Immunotherapy may meet the requirement owing to its antileukemic mechanisms different from those of chemotherapy. Therefore, after reducing tumor burden by chemotherapy, immunotherapy is expected to be a suitable treatment modality to eliminate minimally residual leukemic cells resistant to chemotherapy.

### 2.2. Treatment of Elderly Patients with AML

AML most often occurs in elderly people. It has been reported that 2-year overall survival rate of elderly patients with AML is only 6% [2]. The main reasons of this poor outcome are that AML in elderly patients is often more resistant to chemotherapy than that in younger patients and that elderly patients are intolerable to intensive chemotherapy and allogeneic transplantation. The major advantage of immunotherapy is mild adverse events, and this makes immunotherapy a suitable treatment option for elderly patients.

## 3. AML-Associated Antigens

Recent studies have identified several promising AML antigens suitable for targets of immunotherapy.

Wilms' tumor 1 (WT1) is one of the most promising AML-associated antigens. It was originally reported that HLA-A^*∗*^2402- [3] and HLA-A^*∗*^0201- [4] restricted WT1 peptides induce cytotoxic T lymphocytes (CTLs) that kill WT1-expressing leukemic cells but not normal progenitor cells. WT1 is a transcription factor that plays an important role in leukemogenesis [5], and thus it is less probable that the expression of WT1 is lost. Notably, it has been repeatedly reported that immune responses against WT1 are naturally elicited in cancer patients [6–10], indicating that WT1 protein is immunogenic. These properties render WT1 highly attractive as a tumor antigen.

Proteinase 3 is a myeloid cell-restricted serine protease abundantly expressed in azurophilic granules and is another promising myeloid leukemia-associated antigen. It was originally reported that HLA-A^*∗*^0201-restricted proteinase 3 peptides induce CTLs that preferentially kill myeloid leukemia cells compared to normal marrow cells [11]. Proteinase 3 has also been shown to be immunogenic, as proteinase-3-specific CTLs are induced in a substantial fraction of myeloid leukemia patients in vivo [8, 9, 12]. 

Other than WT1 and proteinase 3, the receptor for hyaluronic-acid-mediated motility (RHAMM/CD168) [13], human telomerase reverse transcriptase (hTERT) [14], preferentially expressed antigen in melanoma (PRAME) [15, 16], and Aurora-A [17] have been reported as potentially useful AML-associated antigens. Notably, WT1 [18] and Aurora-A [17] are reported to be expressed in leukemia stem cells and may thus be suitable targets to eradicate AML.

## 4. Methods of Antigen-Specific Immunotherapy for AML: Active Immunization

Antigen-specific immunotherapies can be largely divided into two categories: active immunization and adoptive T cell therapy. In active immunization, tumor antigens are injected in order to provoke antigen-specific immune responses in vivo. To do so, there are mainly the following three ways reported for AML: peptide vaccines, granulocyte-macrophage colony-stimulating-factor- (GM-CSF) secreting tumor vaccines, and dendritic cell (DC) vaccines. 

### 4.1. Peptide Vaccines

Peptides in combination with an appropriate adjuvant are injected to stimulate CD8+ CTLs specific to the MHC-class-I-restricted peptides. WT1 peptide vaccines have been actively pursued. Oka et al. have first reported a clinical trial of HLA-A^*∗*^2402-restricted WT1 peptide vaccination for malignancies including 12 AML patients [19]. Among 8 patients with evaluable disease, 5 patients achieved decreases in their AML. Notably, in two myelodysplastic syndrome (MDS) patients, numbers of leukocytes in peripheral blood, the majority of which was likely to derive from MDS clones, precipitously decreased after the first administration of the WT1 peptide [20]. This implies remarkable antitumor immune responses in these patients.

Keilholz et al. reported a clinical trial of HLA-A^*∗*^0201-restricted WT1 peptide vaccination for 19 AML or MDS patients, most of whom had large tumor burden [21]. They observed clinical benefit, that is, stable disease or better, in 14 patients and increases in WT1 tetramer+ T cells in blood in 8 patients. Intriguingly, 4 patients had clinical benefit after initial progression, illustrating the importance of evaluating clinical responses to tumor vaccines at later time points even in the presence of initial progression.

Rezvani et al. reported a clinical trial of combined administration of HLA-A^*∗*^0201-restricted WT1 and proteinase 3 peptides to 8 patients with myeloid malignancies [22]. Immune responses to both WT1 and proteinase 3 were detected after a single vaccination in all the patients, suggesting expansion of preexisting memory CD8+ T cells. However, the responses were short-lived and became undetectable after 4 weeks, indicating the necessity of repetitive boost injection.

Maslak et al. reported a clinical trial of a novel combination of WT1 peptide vaccination for 9 AML patients [23]. They used a mixture of 4 peptides; one is an HLA-A^*∗*^0201-restricted heteroclitic peptide that has higher affinity to the HLA class I molecule than a native peptide, and three are long peptides that bind to multiple HLA-DRB1 haplotypes [24]. Interestingly, one of the long peptides embeds the HLA-A^*∗*^0201-restricted heteroclitic peptide in it. The combination of peptides has three potential advantages. First, the heteroclitic peptide is expected to stimulate low avidity tumor-specific CD8+ T cells that preferentially remain in cancer patients. Second, the MHC-class-II-binding peptides can exploit CD4+ T cell help that is required to induce a robust memory CD8+ T cell response. Third, it has been reported that a long peptide containing an MHC-class-I-restricted peptide can be preferentially targeted to professional antigen-presenting DCs that are capable of presenting the embedded MHC-class-I-restricted peptide for a long period [25]. This may avoid suboptimal stimulation of CD8+ T cells resulting from administration of a short peptide that is nonselectively presented by nonprofessional antigen-presenting cells [26]. It is difficult to judge whether this theoretically advantageous strategy leads to a better clinical outcome from the small scale of the study. However, the authors observed the induction of immune responses in 8 out of 9 patients, warranting further study in a larger clinical trial.

Schmitt et al. reported a clinical trial of the HLA-A2-restricted RHAMM peptide vaccination in patients with AML, MDS, and multiple myeloma [27]. In 7 of 10 patients, RHAMM-specific immune responses were detected. Three of 6 patients with myeloid disorders (1/3 AML, 2/3 MDS) achieved clinical responses. This study indicates that RHAMM constitutes a promising target for immunotherapy of AML.

Collectively, these clinical trials of peptide vaccines have established safety and immunogenicity of this modality. Efforts to improve clinical efficacy by combining with superior adjuvants or with other therapeutic modalities will increase the potential of peptide vaccines for AML.

### 4.2. GM-CSF-Secreting Tumor Vaccines

Random mutations in tumor cells are expected to generate many individually specific antigens that may induce multivalent antitumor immune responses of both CD4+ and CD8+ T cells. Thus, the whole autologous tumor cell vaccination is a viable option as long as a sufficient number of tumor cells are harvested in advance.

A mixture of killed autologous leukemia cells and a GM-CSF gene-transduced K562 leukemia cell line was used for vaccination in combination with primed T cells after autologous stem cell transplantation for 54 patients with AML [28]. Leukemic cells are expected to be incorporated into DCs activated by GM-CSF in vivo, and the DCs stimulate antigen-specific T cells. Induction of delayed-type hypersensitivity reactions to autologous tumor cells was associated with 3-year relapse free survival, suggesting a correlation between an immune response and a clinical outcome.

Autologous leukemia cells transduced with GM-CSF were administered after allogeneic transplantation to 28 patients with AML or high-risk MDS [29]. Vaccination elicited local and systemic immune responses despite the administration of a calcineurin inhibitor as prophylaxis against GVHD. Whereas the incidence of GVHD did not increase by vaccination, 9 of 10 patients achieved durable complete remission. Thus, this immunotherapy may potentiate GVL reaction without causing GVHD.

### 4.3. DC Vaccines

DCs generated ex vivo from monocytes or CD34+ progenitor cells are modified to present tumor antigens and are injected. It has also been reported that AML cells can be differentiated into DCs and they can be injected.

In the first DC vaccination for AML, Fujii et al. used CD34+ progenitor cell-derived DCs pulsed with autologous leukemic cells in combination with primed T cells for 4 relapsed patients after allogeneic stem cell transplantation [30]. This method induced tumor-specific immune responses. However, most of the later studies used monocytes as a source of DCs, mainly because it is technically easier to obtain DCs for vaccination from monocytes than CD34+ progenitor cells.

Clinical trials of DC-based immunotherapy for AML using leukemic cell-derived DCs have also been reported [31, 32]. However, the generation of leukemic cell-derived DCs was feasible only in a limited number of patients, and, even in patients with successful generation and vaccinations of leukemic cell-derived DCs, the DC vaccinations could not induce clinically relevant immune responses [32]. This may be due to lower immunostimulatory activity of leukemic cell-derived DCs compared with monocyte-derived DCs (MoDCs) [33], providing a rationale for the use of MoDCs in immunotherapy for AML.

Lee et al. reported the first study of MoDC-based immunotherapy for 2 AML patients with relapse after autologous peripheral blood stem cell transplantation [34]. Although immune responses were induced, the diseases progressed possibly because of high tumor burden before vaccination. In contrast, Van Tendeloo et al. recently reported immunotherapy for AML, 8 patients at complete remission and 2 at partial remission [35]. MoDCs transfected with WT1 mRNA were administered, and molecular remission was achieved in 4 patients including the 2 patients of partial remission. Clinical responses were correlated with increases in WT1-specific CD8+ T cells. This study indicates that vaccination with WT1 mRNA-loaded MoDCs as a postremission treatment may prevent full relapse.

We recently reported two clinical studies of MoDC-based immunotherapy for AML at morphologic complete remission in elderly patients. In one study, we administered MoDCs that engulfed autologous apoptotic leukemic cells to 4 patients [36]. We observed immune responses in 2 patients who exhibited disease stabilization. WT1- and hTERT-specific CD8+ T cell responses were observed in an HLA-A^*∗*^2402-positive patient, indicating cross-priming in vivo. In another study, we administered MoDCs pulsed with an HLA-A^*∗*^2402-restricted modified WT1 peptide that has higher affinity to the HLA molecule than the natural peptide to 3 patients [37]. We observed immune responses in 2 patients who exhibited transient disease stabilization. Notably, CD8+ T cells reactive to the WT1 natural peptide but not to the modified peptide persisted after terminating vaccination, implying that the natural peptide-reactive T cells survived due to stimulation by endogenous cognate antigens.

Collectively, these studies indicate that MoDC-based immunotherapy is immunogenic even in elderly patients with AML after remission-inducing chemotherapy and warrant further study of this strategy.

## 5. Methods of Antigen-Specific Immunotherapy for AML: Adoptive T Cell Therapy

Active immunization relies on immune competence of cancer patients. However, tumor antigen-specific T cells may be nonfunctional or deleted in the presence of tumor cells in vivo in cancer patients [38]. In addition, chemotherapy and immunosuppressive factors from tumor cells may undermine antitumor immunity in cancer patients [39]. Based on these ideas, adoptive transfer of tumor-specific T cells is actively pursued.

Tumor-specific adoptive T cell therapy was initially developed by expanding tumor-infiltrating lymphocytes from melanoma lesions in vitro [40]. However, complicated procedures and difficulty in timely preparation of a sufficient number of cytotoxic T lymphocytes (CTLs) preclude generalization of this strategy. To overcome these drawbacks, adoptive T cell therapies using genetically engineered T cells have recently been prevailing. There are two measures: (i) CD8+ T cells transduced with genes encoding T cell receptor (TCR) that recognizes the complex of a tumor peptide and a particular MHC class I molecule and (ii) T cells transfected with genes encoding chimeric antigen receptor (CAR) that is composed of antibody and cytoplasmic domain of the CD3 molecule. 

### 5.1. Adoptive Transfer of T Cells with Transgenic TCR

The first clinical trial of TCR-transduced T cell transfer was performed to advanced melanoma patients by Rosenberg's group, using HLA-A^*∗*^0201-restricted MART-1, gp100, NY-ESO-1, and p53 as targeted antigens [41]. The transduced T cells were administered after lymphodepleting regimen of fludarabine and cyclophosphamide. Two out of 17 patients achieved partial remission. The absence of therapeutic effects in most cases may be related to the failure of the infused cells to accumulate into the tumor or to exert their effector function in the immunosuppressive tumor microenvironment.

Subsequently, the same group reported a clinical trial using high avidity TCR against HLA-A^*∗*^0201-restricted MART-1 and gp100 peptides [42]. Objective cancer regressions were observed in 30% of patients. Gene-engineered cells persisted at high levels in the blood of all patients 1 month after treatment. However, patients exhibited destruction of normal melanocytes in the skin, eye, and ear. In another study by the same group, a retrovirus encoding the high avidity murine CEA-reactive TCR was used to transduce peripheral blood lymphocytes from 3 HLA-A^*∗*^0201+ patients with metastatic colorectal cancer [43]. All patients experienced profound decreases in serum CEA levels. However, a severe transient inflammatory colitis was induced in all 3 patients. These studies indicate excellent antitumor activity as well as destructive power of highly avid T cells against normal tissues, suggesting the importance of careful assessment of possible damage to normal tissues that share the target antigen with tumor cells.

These promising results of adoptive transfer of TCR-transduced T cells for solid tumors pave the way for its application to hematological malignancies. Two groups reported mouse models of adoptive transfer of T cells with WT1-specific TCR genes [44, 45]. Both of the groups recently reported in vivo therapies for AML using mouse xenograft models transferred with WT1 TCR-transduced T cells [46, 47].

In the T cells transduced with a new TCR gene, their original TCRs are still functional, and thus mispairing of endogenous and introduced TCR chains occurs. This decreases the expression level of introduced TCR, resulting in reduced antitumor activity [48]. In addition, studies in murine models with TCR gene transfer have shown that the mispairing may generate neoreactivity against autoantigens, resulting in GVHD [49]. Ochi et al. circumvented the mispairing problem in an elegant way by developing a novel retroviral vector system for TCR gene transfer that can selectively express target antigen-specific TCR while expression of intrinsic TCRs is suppressed by built-in siRNAs [47, 50]. In a mouse xenograft model, adoptively transferred WT1-siTCR gene-transduced T cells exerted distinct anti-leukemia efficacy, but did not inhibit human hematopoiesis [47]. This is a promising report heading for a clinical trial to treat AML using WT1-TCR T cell transfer.

### 5.2. Adoptive Transfer of T Cells with Transgenic CAR

A CAR contains an extracellular antigen-binding domain, a transmembrane region, and a signaling endodomain. The extracellular domain is typically a single chain variable fragment (scFv) derived from a tumor-specific monoclonal antibody. There are two advantages of using an antibody-derived domain for antigen recognition. First, antibodies are not dependent on MHC presentation. Second, antibodies bind antigens with much greater affinity than TCRs, permitting the formation of a more stable immunological synapse.

CARs can be grouped into three generations with progressively increasing costimulatory activity. These differ primarily in the structure of the signaling endodomain. First-generation CARs contain a single signaling unit derived from the CD3*ζ* chain alone, which transmits a signal inadequate to fully activate T cells. In second-generation CARs, the CD28 intracellular domain is inserted proximal to the CD3*ζ* endodomain to enhance the stimulatory effects of the CAR. This encouraged further addition of other signaling sequences from costimulatory molecules such as 4-1BB and OX40 in third-generation CARs. A complete response was observed in a patient with follicular lymphoma who received T cells transduced with a second-generation anti-CD19 CAR [51]. However, the supraphysiological signal transmitted by second- and third-generation CARs is also a source of concern. In fact, 2 deaths in cancer patients treated with CAR T cells occurred apparently due to cytokine storm: one patient with colon cancer treated with ERBB2-specific CAR [52] and another with chronic lymphocytic leukemia treated with CD19-specific CAR [53]. Although these serious adverse events indeed suggest highly active antitumor effects of CAR, modification to decrease T cell doses and to split infusions will be important to reduce such risk.

The carbohydrate antigen Lewis^Y^ is expressed in about 50% of multiple myeloma and AML cases. Lewis^Y^ CAR-transduced T cells delayed growth of myeloma xenografts in NOD/SCID mice [54]. This paper indicates that Lewis^Y^ CAR T cell transfer is a promising therapy for myeloma and AML.

## 6. Conclusions and Future Prospects

Advances in immunology and identification of promising leukemia-associated antigens are making it possible to develop truly effective immunotherapies for AML. Fortunately, AML is relatively more chemosensitive than most solid tumors, and thus it is possible to reduce a tumor burden by chemotherapy in the majority of patients. Thereafter, immunotherapy will play a complementary role in eradicating minimal residual diseases, which contain chemoresistant leukemia stem cells. Thus, leukemia-associated antigens expressed in leukemia stem cells will be important to achieve cure. 

Recent concerns are immunosuppressive factors expressed by tumor cells or built in the immune system, which curtail antitumor immunity. Universal immunosuppressive factors built in the immune system, such as CTLA-4 [55, 56], PD-1 [57, 58], and regulatory T cells [59–61], are widely applicable targets in combination with antitumor vaccination. However, CTLA-4 blockade caused autoimmune manifestations in considerable fractions of patients [55, 56], which is anticipated from the role of CTLA-4 in maintaining immune homeostasis. Such adverse events, in addition to the autoimmunity [42, 43] and life-threatening cytokine storm [52, 53] observed in the adoptive T cell transfer, indicate that pursuing effectiveness of tumor immunotherapy inherently raises the possibility of harmful immune reactions, if the target antigen is shared by tumor and normal cells or the tumor burden is high. Balance between effectiveness and adverse events will thus become a main issue in the era of advanced immunotherapy. Still, “relative” tumor specificity of immunotherapy, at least, compared to other modalities of cancer therapy will make immunotherapy an indispensable facet of antitumor armamentarium.

Furthermore, epigenetic therapies with DNA methyltransferase and histone deacetylase inhibitors are prevailing as novel therapies for myeloid malignancies. Notably, recent studies have shown that epigenetic modification upregulates the expression of cancer testis antigens in AML and induces CTL responses [62, 63]. This raises possibilities for reasonable combinations of therapies targeting molecular oncogenic pathways and immunotherapies. Such prospects will collectively open an exciting new era of AML therapies in the near future.

## References

[1] Ishikawa F, Yoshida S, Saito Y (2007). Chemotherapy-resistant human AML stem cells home to and engraft within the bone-marrow endosteal region. *Nature Biotechnology*.

[2] Menzin J, Lang K, Earle CC, Kerney D, Mallick R (2002). The outcomes and costs of acute myeloid leukemia among the elderly. *Archives of Internal Medicine*.

[3] Ohminami H, Yasukawa M, Fujita S (2000). HLA class I-restricted lysis of leukemia cells by a CD8+ cytotoxic T- lymphocyte clone specific for WT1 peptide. *Blood*.

[4] Gao L, Bellantuono I, Elsässer A (2000). Selective elimination of leukemic CD34+ progenitor cells by cytotoxic T lymphocytes specific for WT1. *Blood*.

[5] Inoue K, Tamaki H, Ogawa H (1998). Wilms’ tumor gene (WT1) competes with differentiation-inducing signal in hematopoietic progenitor cells. *Blood*.

[6] Gaiger A, Reese V, Disis ML, Cheever MA (2000). Immunity to WT1 in the animal model and in patients with acute myeloid leukemia. *Blood*.

[7] Elisseeva OA, Oka Y, Tsuboi A (2002). Humoral immune responses against Wilms tumor gene WT1 product in patients with hematopoietic malignancies. *Blood*.

[8] Scheibenbogen C, Letsch A, Thiel E (2002). CD8 T-cell responses to Wilms tumor gene product WT1 and proteinase 3 in patients with acute myeloid leukemia. *Blood*.

[9] Rezvani K, Grube M, Brenchley JM (2003). Functional leukemia-associated antigen-specific memory CD8+ T cells exist in healthy individuals and in patients with chronic myelogenous leukemia before and after stem cell transplantation. *Blood*.

[10] Rezvani K, Yong ASM, Savani BN (2007). Graft-versus-leukemia effects associated with detectable Wilms tumor-1-specific T lymphocytes after allogeneic stem-cell transplantation for acute lymphoblastic leukemia. *Blood*.

[11] Molldrem J, Dermime S, Parker K (1996). Targeted T-cell therapy for human leukemia: cytotoxic T lymphocytes specific for a peptide derived from proteinase 3 preferentially lyse human myeloid leukemia cells. *Blood*.

[12] Molldrem JJ, Lee PP, Wang C (2000). Evidence that specific T lymphocytes may participate in the elimination of chronic myelogenous leukemia. *Nature Medicine*.

[13] Greiner J, Li L, Ringhoffer M (2005). Identification and characterization of epitopes of the receptor for hyaluronic acid-mediated motility (RHAMM/CD168) recognized by CD8+ T cells of HLA-A2-positive patients with acute myeloid leukemia. *Blood*.

[14] Arai J, Yasukawa M, Ohminami H, Kakimoto M, Hasegawa A, Fujita S (2001). Identification of human telomerase reverse transcriptase-derived peptides that induce HLA-A24-restricted antileukemia cytotoxic T lymphocytes. *Blood*.

[15] Morita Y, Heike Y, Kawakami M (2006). Monitoring of WT1-specific cytotoxic T lymphocytes after allogeneic hematopoietic stem cell transplantation. *International Journal of Cancer*.

[16] Rezvani K, Yong ASM, Tawab A (2009). Ex vivo characterization of polyclonal memory CD8+ T-cell responses to PRAME-specific peptides in patients with acute lymphoblastic leukemia and acute and chronic myeloid leukemia. *Blood*.

[17] Ochi T, Fujiwara H, Suemori K (2009). Aurora-A kinase: a novel target of cellular immunotherapy for leukemia. *Blood*.

[18] Saito Y, Kitamura H, Hijikata A (2010). Identification of therapeutic targets for quiescent, chemotherapy-resistant human leukemia stem cells. *Science Translational Medicine*.

[19] Oka Y, Tsuboi A, Taguchi T (2004). Induction of WT1 (Wilms’ tumor gene)-specific cytotoxic T lymphocytes by WT1 peptide vaccine and the resultant cancer regression. *Proceedings of the National Academy of Sciences of the United States of America*.

[20] Oka Y, Tsuboi A, Murakami M (2003). Wilms tumor gene peptide-based immunotherapy for patients with overt leukemia from myelodysplastic syndrome (MDS) or MDS with myelofibrosis. *International Journal of Hematology*.

[21] Keilholz U, Letsch A, Busse A (2009). A clinical and immunologic phase 2 trial of Wilms tumor gene product 1 (WT1) peptide vaccination in patients with AML and MDS. *Blood*.

[22] Rezvani K, Yong ASM, Mielke S (2008). Leukemia-associated antigen-specific T-cell responses following combined PR1 and WT1 peptide vaccination in patients with myeloid malignancies. *Blood*.

[23] Maslak PG, Dao T, Krug LM (2010). Vaccination with synthetic analog peptides derived from WT1 oncoprotein induces T-cell responses in patients with complete remission from acute myeloid leukemia. *Blood*.

[24] May RJ, Dao T, Pinilla-Ibarz J (2007). Peptide epitopes from the Wilms’ tumor 1 oncoprotein stimulate CD4 + and CD8+ T cells that recognize and kill human malignant mesothelioma tumor cells. *Clinical Cancer Research*.

[25] Melief CJM, Van Der Burg SH (2008). Immunotherapy of established (pre)malignant disease by synthetic long peptide vaccines. *Nature Reviews Cancer*.

[26] Bijker MS, van den Eeden SJF, Franken KL, Melief CJM, van der Burg SH, Offringa R (2008). Superior induction of anti-tumor CTL immunity by extended peptide vaccines involves prolonged, DC-focused antigen presentation. *European Journal of Immunology*.

[27] Schmitt M, Schmitt A, Rojewski MT (2008). RHAMM-R3 peptide vaccination in patients with acute myeloid leukemia, myelodysplastic syndrome, and multiple myeloma elicits immunologic and clinical responses. *Blood*.

[28] Borrello IM, Levitsky HI, Stock W (2009). Granulocyte-macrophage colony-stimulating factor (GM-CSF)-secreting cellular immunotherapy in combination with autologous stem cell transplantation (ASCT) as postremission therapy for acute myeloid leukemia (AML). *Blood*.

[29] Ho VT, Vanneman M, Kim H (2009). Biologic activity of irradiated, autologous, GM-CSF-secreting leukemia cell vaccines early after allogeneic stem cell transplantation. *Proceedings of the National Academy of Sciences of the United States of America*.

[30] Fujii SI, Shimizu K, Fujimoto K (2001). Treatment of post-transplanted, relapsed patients with hematological malignancies by infusion of HLA-matched, allogeneic-dendritic cells (DCs) pulsed with irradiated tumor cells and primed T cells. *Leukemia and Lymphoma*.

[31] Li L, Giannopoulos K, Reinhardt P (2006). Immunotherapy for patients with acute myeloid leukemia using autologous dendritic cells generated from leukemic blasts. *International Journal of Oncology*.

[32] Roddie H, Klammer M, Thomas C (2006). Phase I/II study of vaccination with dendritic-like leukaemia cells for the immunotherapy of acute myeloid leukaemia. *The British Journal of Haematology*.

[33] Draube A, Beyer M, Wolf J (2008). Activation of autologous leukemia-specific T cells in acute myeloid leukemia: monocyte-derived dendritic cells cocultured with leukemic blasts compared with leukemia-derived dendritic cells. *European Journal of Haematology*.

[34] Lee JJ, Kook H, Park MS (2004). Immunotherapy using autologous monocyte-derived dendritic cells pulsed with leukemic cell lysates for acute myeloid leukemia relapse after autologous peripheral blood stem cell transplantation. *Journal of Clinical Apheresis*.

[35] Van Tendeloo VF, Van De Veldea A, Van Driesschea A (2010). Induction of complete and molecular remissions in acute myeloid leukemia by Wilms’ tumor 1 antigen-targeted dendritic cell vaccination. *Proceedings of the National Academy of Sciences of the United States of America*.

[36] Kitawaki T, Kadowaki N, Fukunaga K (2011). Cross-priming of CD8+ T cells in vivo by dendritic cells pulsed with autologous apoptotic leukemic cells in immunotherapy for elderly patients with acute myeloid leukemia. *Experimental Hematology*.

[37] Kitawaki T, Kadowaki N, Fukunaga K (2011). A phase I/IIa clinical trial of immunotherapy for elderly patients with acute myeloid leukaemia using dendritic cells co-pulsed with WT1 peptide and zoledronate. *The British Journal of Haematology*.

[38] Theobald M, Biggs J, Hernández J, Lustgarten J, Labadie C, Sherman LA (1997). Tolerance to p53 by A2.1-restricted cytotoxic T lymphocytes. *Journal of Experimental Medicine*.

[39] Finn OJ (2008). Molecular origins of cancer: cancer immunology. *The New England Journal of Medicine*.

[40] Rosenberg SA, Yannelli JR, Yang JC (1994). Treatment of patients with metastatic melanoma with autologous tumor-infiltrating lymphocytes and interleukin 2. *Journal of the National Cancer Institute*.

[41] Morgan RA, Dudley ME, Wunderlich JR (2006). Cancer regression in patients after transfer of genetically engineered lymphocytes. *Science*.

[42] Johnson LA, Morgan RA, Dudley ME (2009). Gene therapy with human and mouse T-cell receptors mediates cancer regression and targets normal tissues expressing cognate antigen. *Blood*.

[43] Parkhurst MR, Yang JC, Langan RC (2010). T cells targeting carcinoembryonic antigen can mediate regression of metastatic colorectal cancer but induce severe transient colitis. *Molecular Therapy*.

[44] Tsuji T, Yasukawa M, Matsuzaki J (2005). Generation of tumor-specific, HLA class I-restricted human Th1 and Tc1 cells by cell engineering with tumor peptide-specific T-cell receptor genes. *Blood*.

[45] Xue SA, Gao L, Hart D (2005). Elimination of human leukemia cells in NOD/SCID mice by WT1-TCR gene-transduced human T cells. *Blood*.

[46] Xue SA, Gao L, Thomas S (2010). Development of a Wilms’ tumor antigen-specific T-cell receptor for clinical trials: engineered patient’s T cells can eliminate autologous leukemia blasts in NOD/SCID mice. *Haematologica*.

[47] Ochi T, Fujiwara H, Okamoto S (2011). Novel adoptive T-cell immunotherapy using a WT1-specific TCR vector encoding silencers for endogenous TCRs shows marked anti-leukemia reactivity and safety. *Blood*.

[48] Schmitt TM, Ragnarsson GB, Greenberg PD (2009). T cell receptor gene therapy for cancer. *Human Gene Therapy*.

[49] Bendle GM, Linnemann C, Hooijkaas AI (2010). Lethal graft-versus-host disease in mouse models of T cell receptor gene therapy. *Nature Medicine*.

[50] Okamoto S, Mineno J, Ikeda H (2009). Improved expression and reactivity of transduced tumor-specific TCRs in human lymphocytes by specific silencing of endogenous TCR. *Cancer Research*.

[51] Kochenderfer JN, Wilson WH, Janik JE (2010). Eradication of B-lineage cells and regression of lymphoma in a patient treated with autologous T cells genetically engineered to recognize CD19. *Blood*.

[52] Morgan RA, Yang JC, Kitano M, Dudley ME, Laurencot CM, Rosenberg SA (2010). Case report of a serious adverse event following the administration of t cells transduced with a chimeric antigen receptor recognizing ERBB2. *Molecular Therapy*.

[53] Brentjens R, Yeh R, Bernal Y, Riviere I, Sadelain M (2010). Treatment of chronic lymphocytic leukemia with genetically targeted autologous t cells: case report of an unforeseen adverse event in a phase i clinical trial. *Molecular Therapy*.

[54] Peinert S, Prince HM, Guru PM (2010). Gene-modified T cells as immunotherapy for multiple myeloma and acute myeloid leukemia expressing the Lewis y antigen. *Gene Therapy*.

[55] Hodi FS, O’Day SJ, McDermott DF (2010). Improved survival with ipilimumab in patients with metastatic melanoma. *The New England Journal of Medicine*.

[56] Phan GQ, Yang JC, Sherry RM (2003). Cancer regression and autoimmunity induced by cytotoxic T lymphocyte-associated antigen 4 blockade in patients with metastatic melanoma. *Proceedings of the National Academy of Sciences of the United States of America*.

[57] Brahmer JR, Drake CG, Wollner I (2010). Phase I study of single-agent anti-programmed death-1 (MDX-1106) in refractory solid tumors: safety, clinical activity, pharmacodynamics, and immunologic correlates. *Journal of Clinical Oncology*.

[58] Iwai Y, Ishida M, Tanaka Y, Okazaki T, Honjo T, Minato N (2002). Involvement of PD-L1 on tumor cells in the escape from host immune system and tumor immunotherapy by PD-L1 blockade. *Proceedings of the National Academy of Sciences of the United States of America*.

[59] Ishida T, Ishii T, Inagaki A (2006). Specific recruitment of CC chemokine receptor 4-positive regulatory T cells in Hodgkin lymphoma fosters immune privilege. *Cancer Research*.

[60] Shimizu J, Yamazaki S, Sakaguchi S (1999). Induction of tumor immunity by removing CD25+CD4+ T cells: a common basis between tumor immunity and autoimmunity. *Journal of Immunology*.

[61] Morse MA, Hobeika AC, Osada T (2008). Depletion of human regulatory T cells specifically enhances antigen-specific immune responses to cancer vaccines. *Blood*.

[62] Almstedt M, Blagitko-Dorfs N, Duque-Afonso J (2010). The DNA demethylating agent 5-aza-2’-deoxycytidine induces expression of NY-ESO-1 and other cancer/testis antigens in myeloid leukemia cells. *Leukemia Research*.

[63] Goodyear O, Agathanggelou A, Novitzky-Basso I (2010). Induction of a CD8+ T-cell response to the MAGE cancer testis antigen by combined treatment with azacitidine and sodium valproate in patients with acute myeloid leukemia and myelodysplasia. *Blood*.

